# Resilience for Juvenile Recidivists Versus One‑Time Offenders in Argentina

**DOI:** 10.1192/j.eurpsy.2023.926

**Published:** 2023-07-19

**Authors:** M. S. S. Orlando

**Affiliations:** Juveniles, National Supreme Court of Argentina, Buenos Aires, Argentina

## Abstract

**Introduction:**

Resilient potential of 100 young male offenders (aged 16-17), in Buenos Aires was assessed using a translated and revised version of the Resilience Scale (RS) of 14 items (Wagnild, 2009). Data on family criminality, school achievement and socioeconomic status was also obtained for both groups. The greater the resilient potential the greater the opportunity of not to reoffend irrespective of being controlled by key risk factors.

**Objectives:**

1-Do repeat offenders have lower resilient potential than one-time offenders? 2. If a relationship between resilience and repeat offending does exist, is this explained by family criminality, low school achievement or low socioeconomic status?

**Methods:**

Psychosocial interviews with each participant were conducted by the named author, under strict judicial conditions considering privacy in all cases, based on informed consent, with the condition of maintaining the concealed identity of the participants. In all cases it was clarified that youth participation was voluntary.

**Results:**

Table 1.

M (SD) M (SD) t d p

Age 16.20 (0.41) 16.45 (0.50) 2.70 0.66 0.008

Resilience M (SD) M (SD) t d p

Factor I 29.92 (7.25) 63.45 (4.92) 27.16 5.41 <0.001

Factor II 7.37 (1.95) 16.59 (1.87) 24.13 11.33 <0.001

Total Resilience 37.29 (8.67) 80.09 (6.49) 27.82 5.59 <0.001

Risk Factors % % chi d p

Family Criminality 44.9 3.9 20.81 1.03 <0.001

Low School Achievement 98.0 11.8 71.31 3.15 <0.001

Low Socioeconomic Status 42.9 11.8 10.73 0.69 0.001

Table 2. Independent Predictors of Repeat Offending

Variables β p

Criminality in the family 2.994 <0.001

Low school achievement 5.886 <0.001

Low socioeconomic status (SES) -1.727 <0.001

Note. All comparisons p<.001.

**Conclusions:**

Taking into account the lack of studies on resilience in juvenile offenders in both national and international research the findings of the present study provide an important contribution in the field of juvenile offending, particularly in the view of further interventions aimed at the prevention and rehabilitation in the near and long term of juvenile offenders in Argentina.

**Disclosure of Interest:**

None Declared

